# Supplementary material for: Arterial Blood Gas Analysis and Clinical Decision-Making in Emergency and Intensive Care Unit Nurses: A Performance Evaluation
Source: Healthcare (Basel). 2025 Jan 28;13(3):261. doi: 10.3390/healthcare13030261 (PMC11816711; doi:10.3390/healthcare13030261)
Supplement: Supplementary file 1 [file healthcare-13-00261-s001.zip › healthcare-3416177-supplementary.pdf]

Case	Clinical History	Vital signs	Arterial BGA
1	83-year-old male. The patient was referred from the day hospital due to asthenia and respiratory difficulties. He reports an increase in abdominal size and peripheral edema. The patient is alert, cooperative, and experiences significant dyspnea with minimal physical exertion.<br>Medical history (APR): atrial fibrillation, hypertension, heart failure, liver tumor.<br>Current therapy: Lasix, Isoptin, Vertiser, Clexane.	HR: 105<br>RR: 20<br>SpO2: 93<br>Temp: 36.0<br>BP: 110/77<br>GCS: 15<br>FiO2: 21	Ph: 7,526<br>pCO_2: 37.7<br>pO_2: 69.4<br>SBE: 8,4<br>HCO_3: 31.6<br>Latt: 2.0<br>Gluc: 133<br>Hb: 14.2

Supplementary File 1: Example of a clinical case presented for nurses: as shown, the clinical case includes clinical history, vital signs and arterial blood gas analysis data.
